# Multifocal Presentations of Myelin Oligodendrocyte Glycoprotein Antibody-Associated Disease (MOGAD): A Case Series From a District General Hospital in South East England

**DOI:** 10.7759/cureus.96706

**Published:** 2025-11-12

**Authors:** Mohamed Saifaldin Eltayieb Mohamed, Doaa Mahmoud Akasha Mohamed, Mok Tzehow, Elin Gurung, Habeeb Jarral

**Affiliations:** 1 Acute Medicine, Surrey and Sussex Healthcare NHS Trust, East Surrey Hospital, Redhill, GBR; 2 Pediatrics, University of Gezira, Wad Madani, SDN; 3 Neurology, Surrey and Sussex Healthcare NHS Trust, East Surrey Hospital, Redhill, GBR; 4 General Internal Medicine, Surrey and Sussex Healthcare NHS Trust, East Surrey Hospital, Redhill, GBR

**Keywords:** anti-mog antibody, bilateral optic perineuritis, longitudinally extensive transverse myelitis (letm), mogad, mog antibody-associated disease (mogad), myelin oligodendrocyte glycoprotein antibody-associated disease, myelin oligodendrocyte glycoprotein antibody-associated disease (mogad), myelitis, neuromyelitis optica spectrum disorder, optic neuritis

## Abstract

This case series describes five patients with diverse clinical presentations of myelin oligodendrocyte glycoprotein (MOG) antibody-associated demyelinating disease. MOG antibody-associated disease (MOGAD) is an autoimmune inflammatory disorder of the central nervous system (CNS), distinct from multiple sclerosis (MS) and aquaporin-4 antibody-positive neuromyelitis optica spectrum disorder (AQP4 Ab+ NMOSD). Our cases highlight the varied manifestations, diagnostic challenges, and treatment responses among patients with MOGAD, emphasizing the importance of early diagnosis and appropriate management to prevent relapses and long-term disability.

## Introduction

Myelin oligodendrocyte glycoprotein (MOG) is a glycoprotein expressed on oligodendrocytes and myelin sheaths in the central nervous system (CNS) [1,2]. Antibodies directed against MOG cause a spectrum of inflammatory demyelinating syndromes collectively termed MOG antibody-associated disease (MOGAD) [1]. MOGAD is a distinct autoimmune disorder separate from multiple sclerosis (MS) and aquaporin-4 antibody-positive neuromyelitis optica spectrum disorder (AQP4 Ab+ NMOSD).

MOGAD has a female-to-male ratio of approximately 1:1, which differs from the female predominance typically observed in other autoimmune or immune-mediated disorders, including AQP4 Ab+ NMOSD (9:1) and MS (3:1) [1]. The relative frequency of MOGAD among demyelinating CNS disorders appears higher in children (<18 years of age) than in adults, although any age can be affected. The overall prevalence of MOGAD is low but increasingly recognized due to improved antibody testing.

Diagnosis combines clinical, radiological, and serological evaluation, with MOG-IgG detection by live cell-based assay (CBA), currently regarded as the gold standard; however, in our case series, antibody testing was performed in an external UK reference laboratory, and the specific assay type was not disclosed. Typical MRI findings include optic neuritis with sheath enhancement, central gray matter longitudinally extensive transverse myelitis (LETM), acute disseminated encephalomyelitis (ADEM) lesions, and brainstem or cerebellar lesions.

MOGAD poses diagnostic challenges due to overlapping features with infectious, postinfectious, and other autoimmune demyelinating syndromes, and misdiagnosis can delay appropriate therapy. Although many patients respond well to corticosteroids and plasma exchange (PLEX), the disease can relapse, particularly in individuals with persistent antibody positivity, highlighting the importance of early recognition and long-term monitoring [1,2].

This single-center case series describes five adults with multifocal MOGAD, defined as the involvement of more than one CNS region either concurrently or sequentially. We emphasize diagnostic pitfalls such as infectious overlap, the utility of rapid antibody testing, and the role of early corticosteroids and plasma exchange in improving neurological outcomes.

## Case presentation

Case 1: Optic neuritis and perineuritis

A 17-year-old right-hand-dominant female patient presented with a two-week history of progressive, painful loss of vision in both eyes (worse on the right than the left), eventually leading to complete visual loss with no perception of light, along with photophobia and bitemporal headache. Two weeks before admission, she experienced a coryzal illness that was treated with amoxicillin.

Clinical Examination and Investigation Findings

Fundoscopy at presentation, before total visual loss, revealed bilateral optic disc swelling, more pronounced in the right eye. Ishihara scores were 0/21 for the right eye (OD) and 18/21 for the left. Visual acuity was 6/60 in the right eye and 6/12 in the left. Other neurological examinations were normal.

CT of the head was normal and did not show any acute intracranial pathology.

In view of the optic disc swelling and normal CT of the head findings, a lumbar puncture was performed to assess cerebrospinal fluid (CSF) characteristics (Table 1).

**Table 1:** Cerebrospinal fluid (CSF) sample showing lymphocytic predominance consistent with a non-bacterial etiology.

Parameter	Result	Reference Range (Normal)	Interpretation
Opening pressure	26.8 cm H₂O	10-20 cm H₂O	Mildly elevated
Appearance	Clear and colorless	Clear and colorless	Normal
Glucose (CSF)	3.1 mmol/L	2.5-4.4 mmol/L (or >50% of serum glucose)	Normal
Serum glucose	5.3 mmol/L	-	-
CSF/serum glucose ratio	0.58	>0.5	Normal
Protein	0.63 g/L	0.15-0.45 g/L	Mildly elevated
Lactate	1.5 mmol/L	1.1-2.4 mmol/L	Normal
White blood cells (WBC)	586 cells/µL	<5 cells/µL	Markedly elevated
Differential count	69% lymphocytes	-	Lymphocytic predominance (suggests viral or inflammatory cause)
Organisms (microscopy)	None seen	None	Normal
Culture	Negative	Negative	Non-bacterial etiology likely

**Figure 1:**
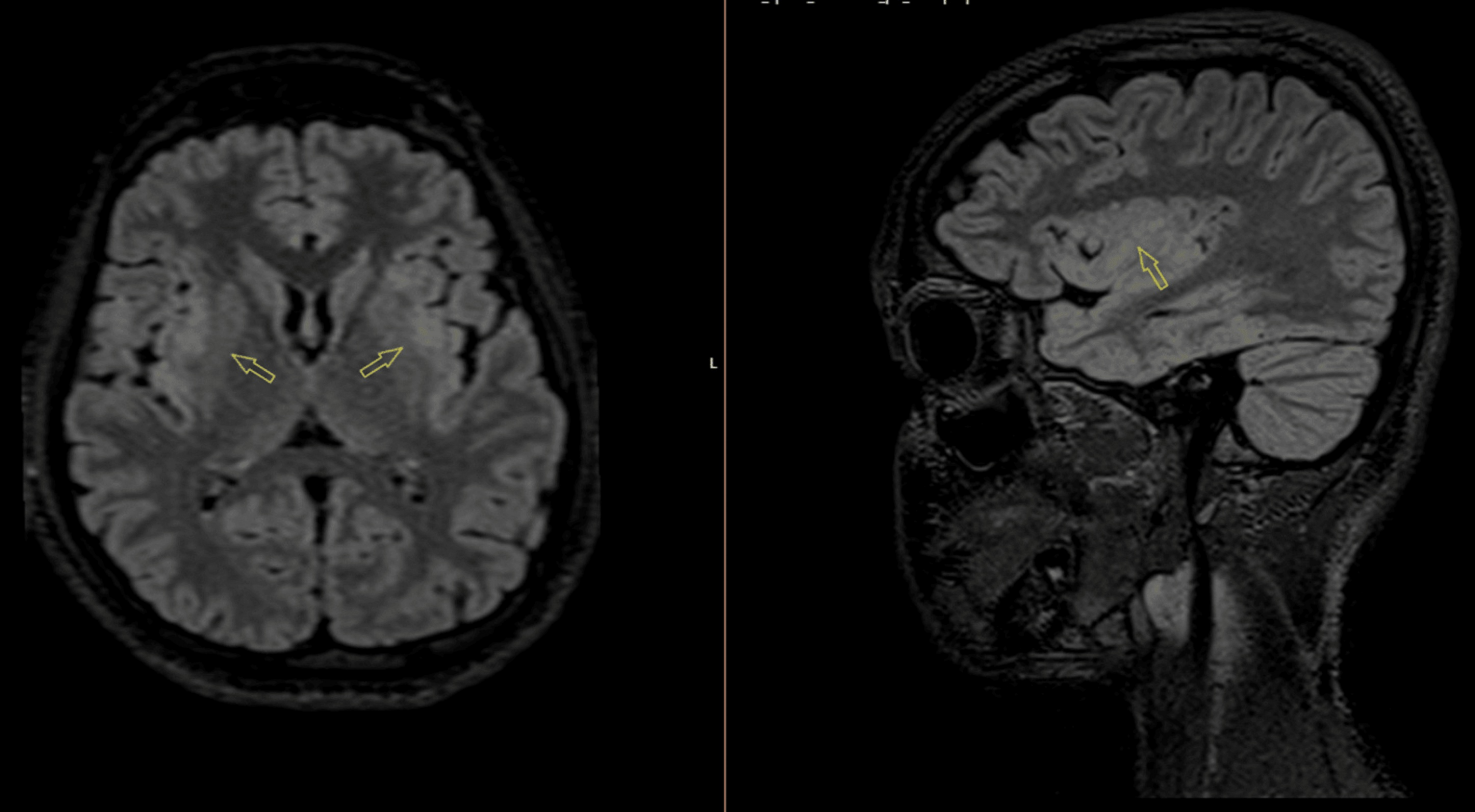
Axial fluid-attenuated inversion recovery (FLAIR) MRI showing swelling (edema) in both insular regions of the brain.

**Figure 2:**
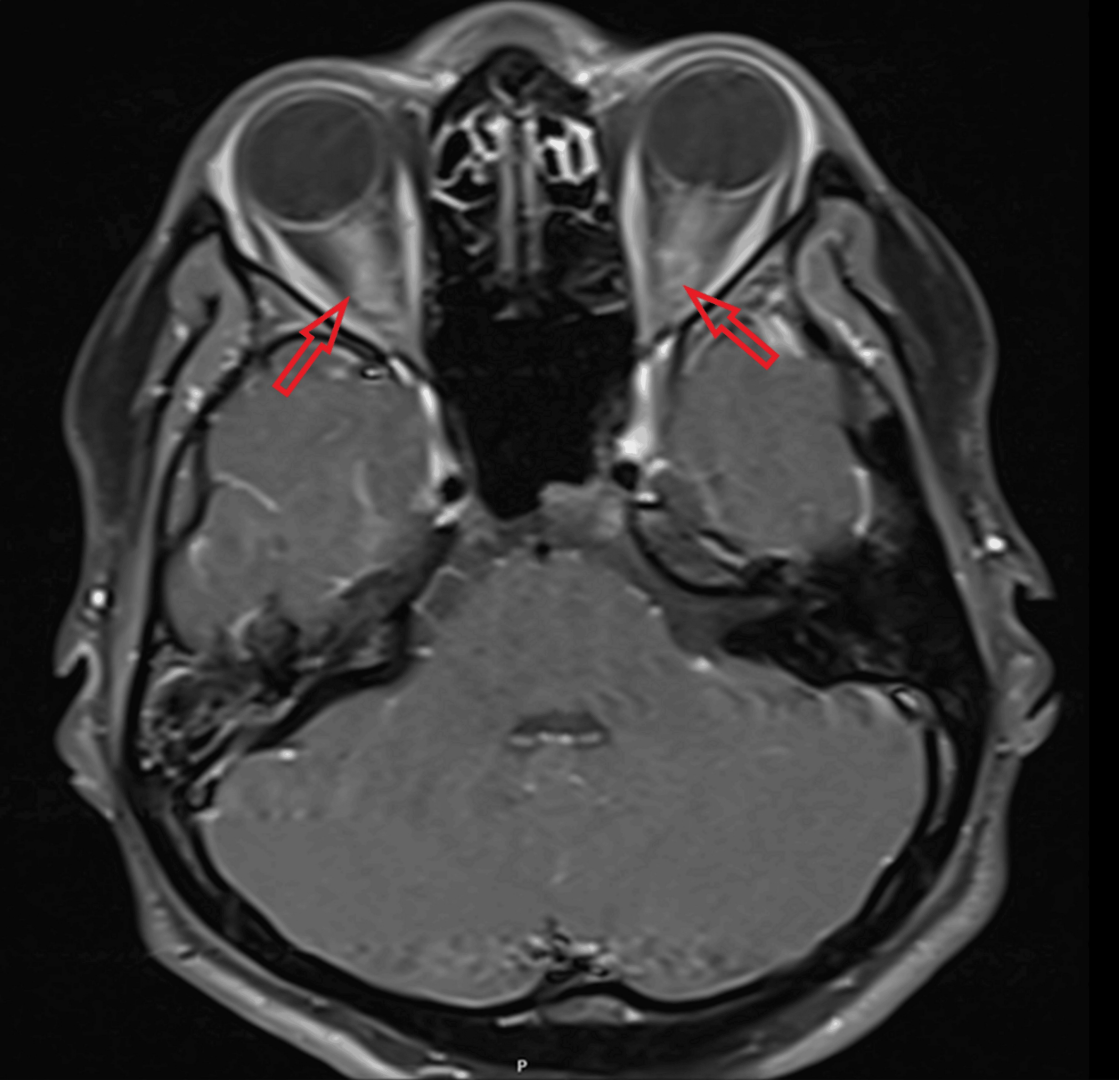
Axial T1 post-contrast MRI showing enhancement (bright signal) around both optic nerve sheaths, indicating inflammation.

CT venogram was unremarkable for dural venous sinus thrombosis but noted narrow bilateral transverse and sigmoid sinuses. Contrast MRI of the brain and orbit showed bilateral insular cortical edema (Figure 1), right-sided temporal and inferior frontal cortical edema, bilateral hippocampal edema (right > left), and a tiny patch of edema in the left medial thalamus. Patches of parenchymal enhancement were seen in the right mesial and inferior frontal lobe, insulae, left frontal lobe, and mesial frontal and temporal leptomeningeal enhancement. Bilateral optic nerve swelling and enhancement, worse on the right, with some stranding in the orbital fat and pre-chiasmatic optic nerve involvement, were also noted. Optic nerve sheath enhancement (perineuritis) was also present (Figure 2).

Diagnosis and Treatment

Due to a high index of suspicion, blood samples were prioritized for expedited turnover for anti-MOG and anti-AQP4 antibodies. Anti-MOG antibodies were positive (titer of 1:1000), while anti-AQP4 antibodies were negative, rapidly confirming a serological diagnosis of anti-MOG antibody-associated optic neuritis and perineuritis. She was treated with intravenous (IV) methylprednisolone 1 g OD for three days, followed by a slowly tapering dose of oral prednisolone 60 mg OD, and was referred for emergency plasma exchange (PLEX) at a tertiary neuroscience referral center.

Outcome and Follow-Up

Following steroid treatment and PLEX, ​​her vision improved dramatically within four weeks of PLEX. A six-month follow-up MRI of the brain showed the resolution of parenchymal FLAIR and T2 signal hyperintensities with no new lesions or abnormal enhancement. The optic nerves appeared normal. Eight months later, anti-MOG antibodies remained positive, necessitating the introduction of a steroid-sparing immunosuppressant; she developed an allergic skin rash on azathioprine but is tolerating mycophenolate mofetil well. She was started on mycophenolate as a steroid-sparing immunosuppressant and continued prednisolone for another six months.

Case 2: Rhomboencephalitis and myelitis

A 27-year-old man from Pakistan, studying in the United Kingdom, with no significant past medical history, presented with a seven-day history of abdominal pain and vomiting.

Clinical Presentation and Investigations

He had multiple ED attendances for urinary retention (requiring catheterization), lower abdominal pain, headache, joint pain, syncope, blurred vision, and fever. He was subsequently admitted with dizziness, fever, tremor, drowsiness, headache, and photophobia.

In light of his neurological presentation, cerebrospinal fluid (CSF) analysis was undertaken to investigate for meningoencephalitis (Table 2).

**Table 2:** Cerebrospinal fluid (CSF) sample showing lymphocytic pleocytosis and elevated protein, consistent with a non-bacterial inflammatory or viral etiology.

Parameter	Result	Reference Range (Normal)	Interpretation
Appearance	Clear and colorless	Clear and colorless	Normal
White blood cells (WBC)	77 cells/µL	<5 cells/µL	Elevated
Differential count	99% lymphocytes	-	Lymphocytic predominance (suggests viral or inflammatory cause)
Protein	1.09 g/L	0.15-0.45 g/L	Markedly elevated
Glucose (CSF)	2.8 mmol/L	2.5-4.4 mmol/L (or >50% of serum glucose)	Normal
Appearance on microscopy	No organisms seen	None	Non-bacterial etiology likely

He rapidly developed tetraparesis, obtundation, and type 1 respiratory failure, necessitating intubation.

**Figure 3:**
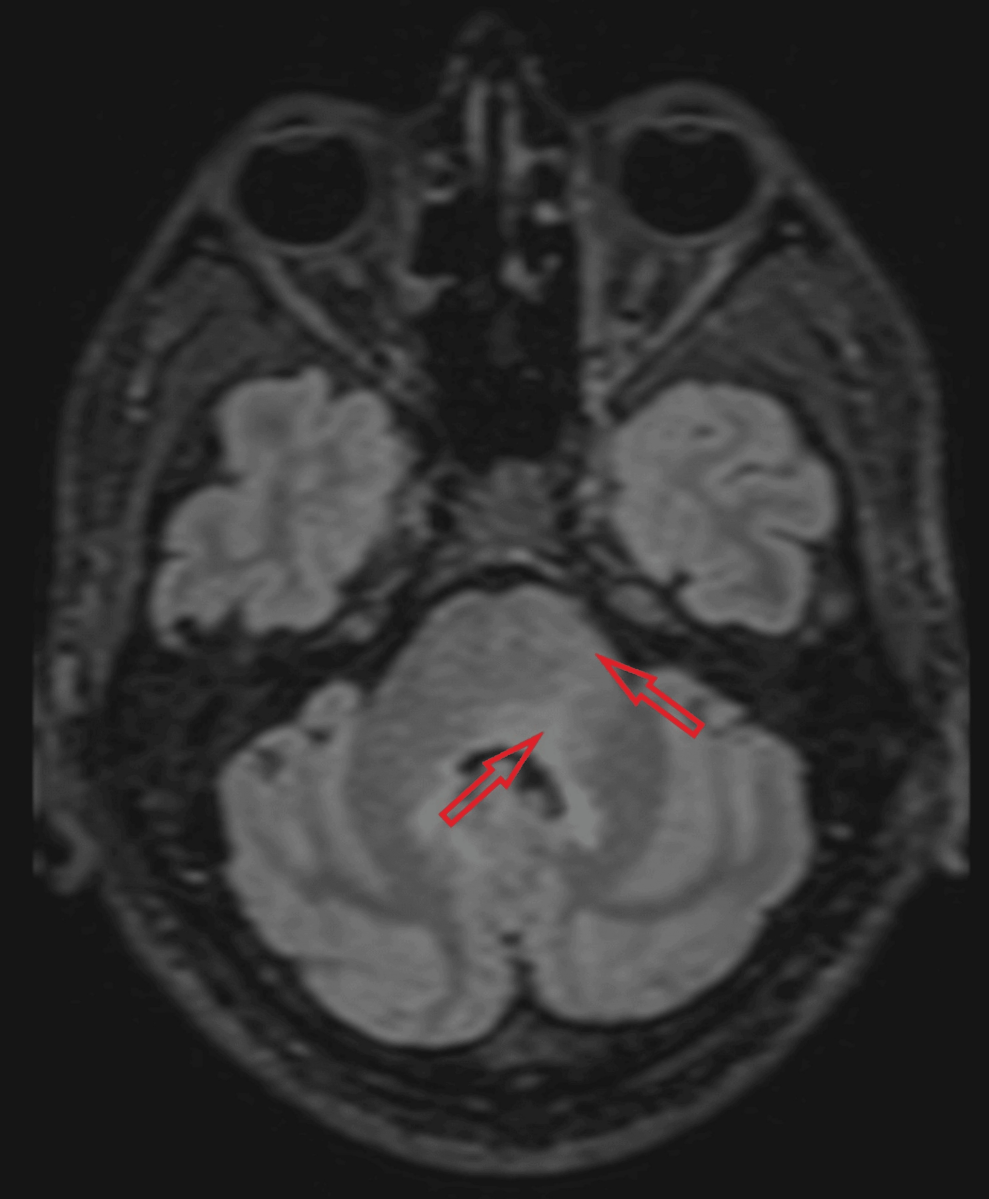
Axial fluid-attenuated inversion recovery (FLAIR) MRI showing patchy signal hyperintensity and edema around the fourth ventricle (left greater than right) and left cerebellar peduncle.

**Figure 4:**
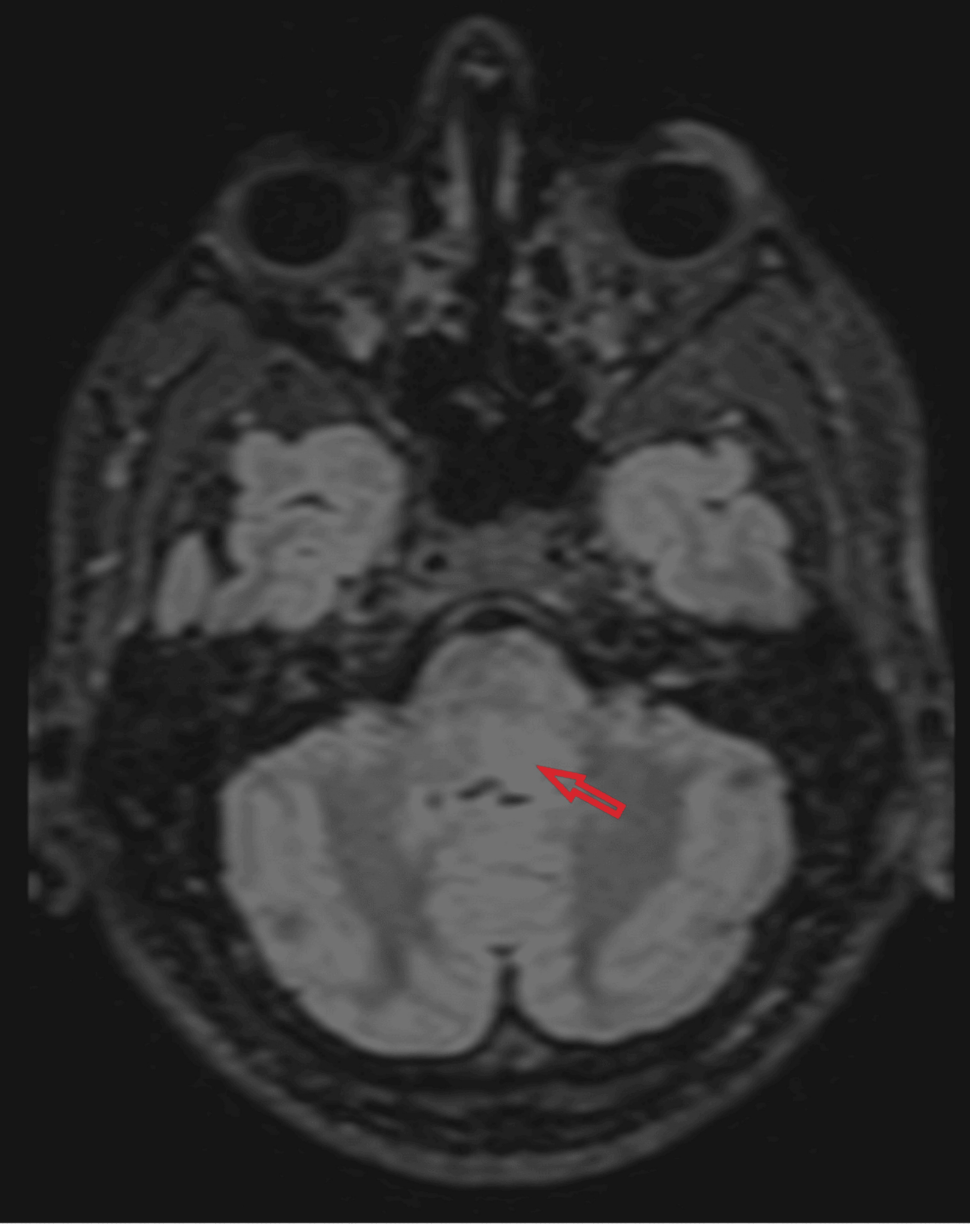
Axial fluid-attenuated inversion recovery (FLAIR) MRI demonstrating signal hyperintensity and edema predominantly in the left dorsal lower pons.

**Figure 5:**
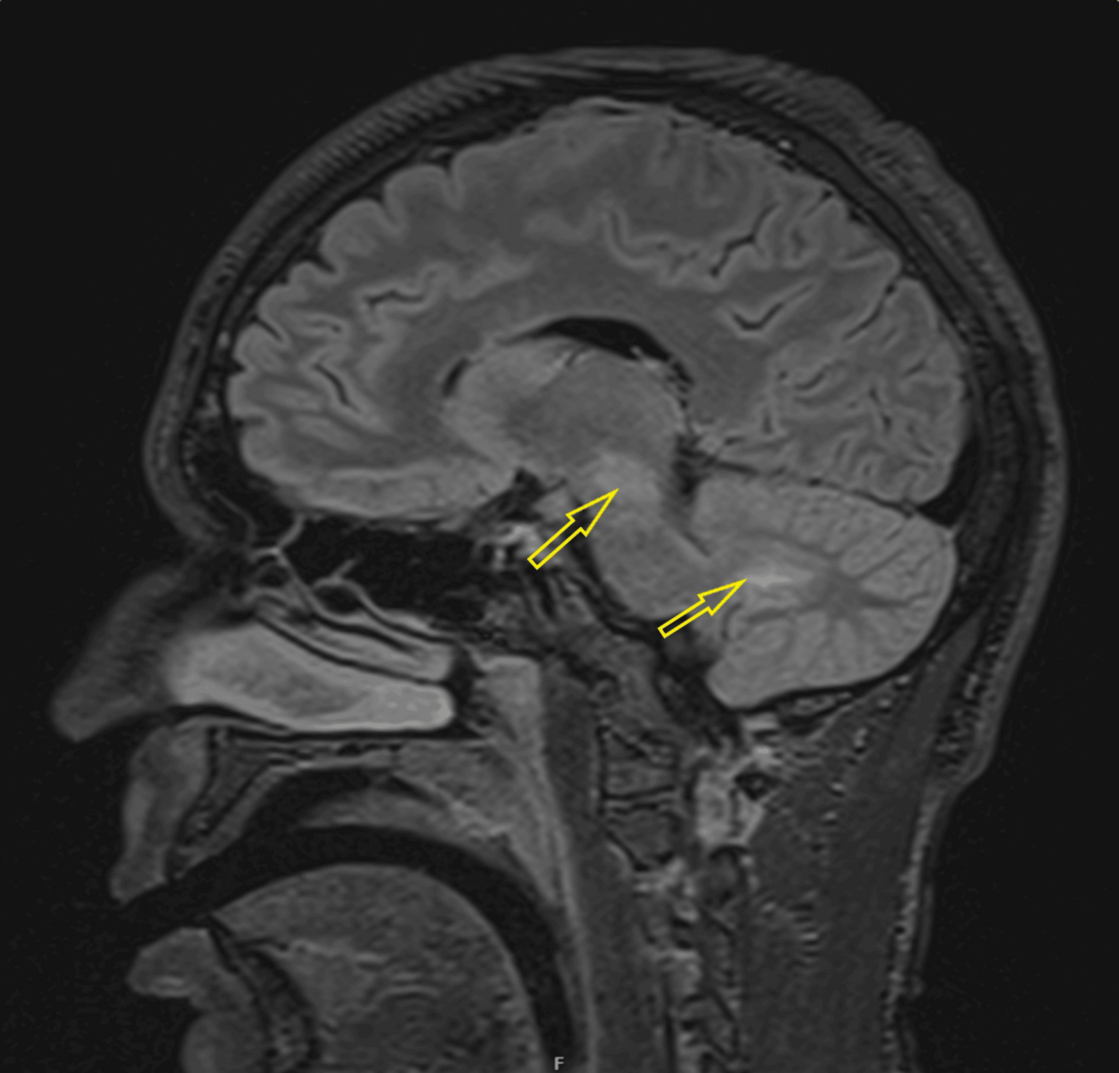
Sagittal fluid-attenuated inversion recovery (FLAIR) MRI demonstrating signal hyperintensity and edema in the dorsal midbrain and cerebellar white matter.

**Figure 6:**
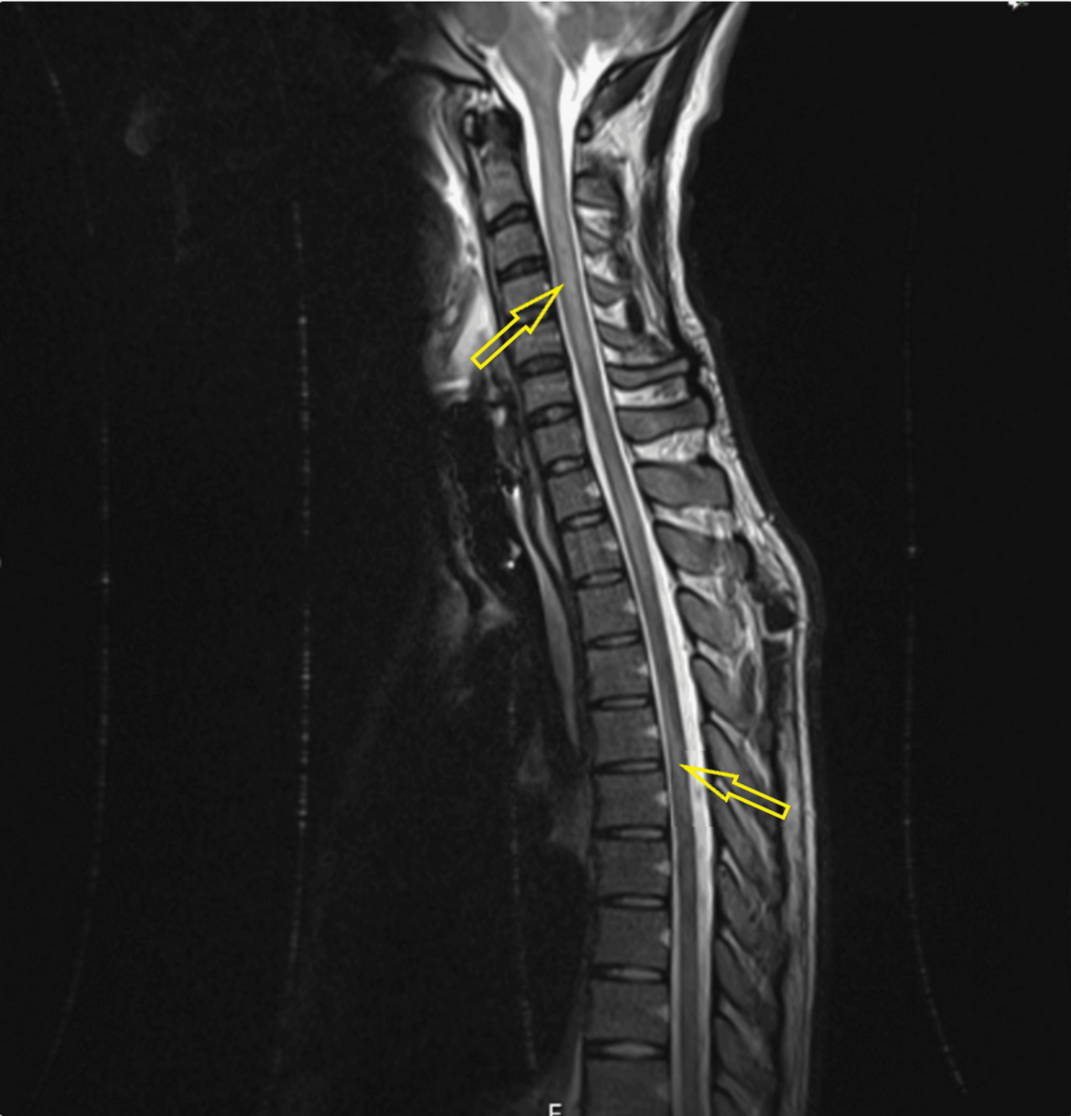
Sagittal T2-weighted MRI showing a long segment of spinal cord inflammation extending from the cervical to thoracic levels (longitudinally extensive transverse myelitis, LETM).

MRI of the head and spine revealed focal edema without restricted diffusion in the midbrain/subthalamic region, dorsal pons, superior cerebellar peduncles, white matter around the fourth ventricle (Figures 3-5), and cervical/thoracic spinal cord (centered on central gray matter). These appearances were consistent with rhombencephalitis and longitudinally extensive transverse myelitis (LETM) (Figure 6).

Diagnosis and Treatment

Differential diagnoses included infective causes (viral, mycoplasma, Lyme disease, and tuberculosis {TB}) and inflammatory etiologies (NMOSD, including AQP4 and MOG antibody-related neuroinflammation) [2-4]. There was some debate as to whether his clinical picture could have been caused by tuberculous meningoencephalomyelitis or neuroinflammatory demyelination. Expedited testing of his serum anti-MOG and anti-AQP4 antibodies revealed a positive result for anti-MOG Ab (1:100). Pulsed methylprednisolone treatment (500 mg daily over five days) was administered, followed by oral prednisolone 60 mg daily, and he was transferred to a tertiary neuroscience center for emergency PLEX. During the convalescent period, he was subsequently discovered to have a positive TB Quantiferon and cervical lymphadenopathy. Biopsy of a cervical lymph node revealed fully sensitive *Mycobacterium tuberculosis*. This was successfully treated with a conventional antituberculous regimen.

Outcome and Follow-Up

A follow-up orbital MRI performed one month later demonstrated normal appearances of the anterior visual pathway, with marked improvement in the brain parenchymal lesions. At six weeks from the initial presentation, dysphagia had improved, and limb tone and reflexes had normalized; he was left with mild erectile and bladder dysfunction. Prednisolone was slowly tapered and fully withdrawn. He continued TB treatment, which was extended for a total of one year due to persistent lymphadenopathy. Repeat anti-MOG antibodies were negative eight months later, indicating a low risk of relapse. One year later, he completed TB treatment and was discharged from the TB services. He remains on annual MOG antibody checks and neurology follow-up.

Case 3: Bilateral optic perineuritis

A 52-year-old man with no significant past medical history presented with a three-day history of progressive, painful bilateral visual loss, associated with fever. Notably, he had received an influenza vaccine a few weeks prior to symptom onset [5].

Clinical Presentation and Investigations

He reported progressive visual loss that was not altitudinal, describing it as an "overexposed whiteout," along with red desaturation. His vision deteriorated to complete blindness in the left eye, while in the right eye, he retained only the ability to perceive hand movements.

**Figure 7:**
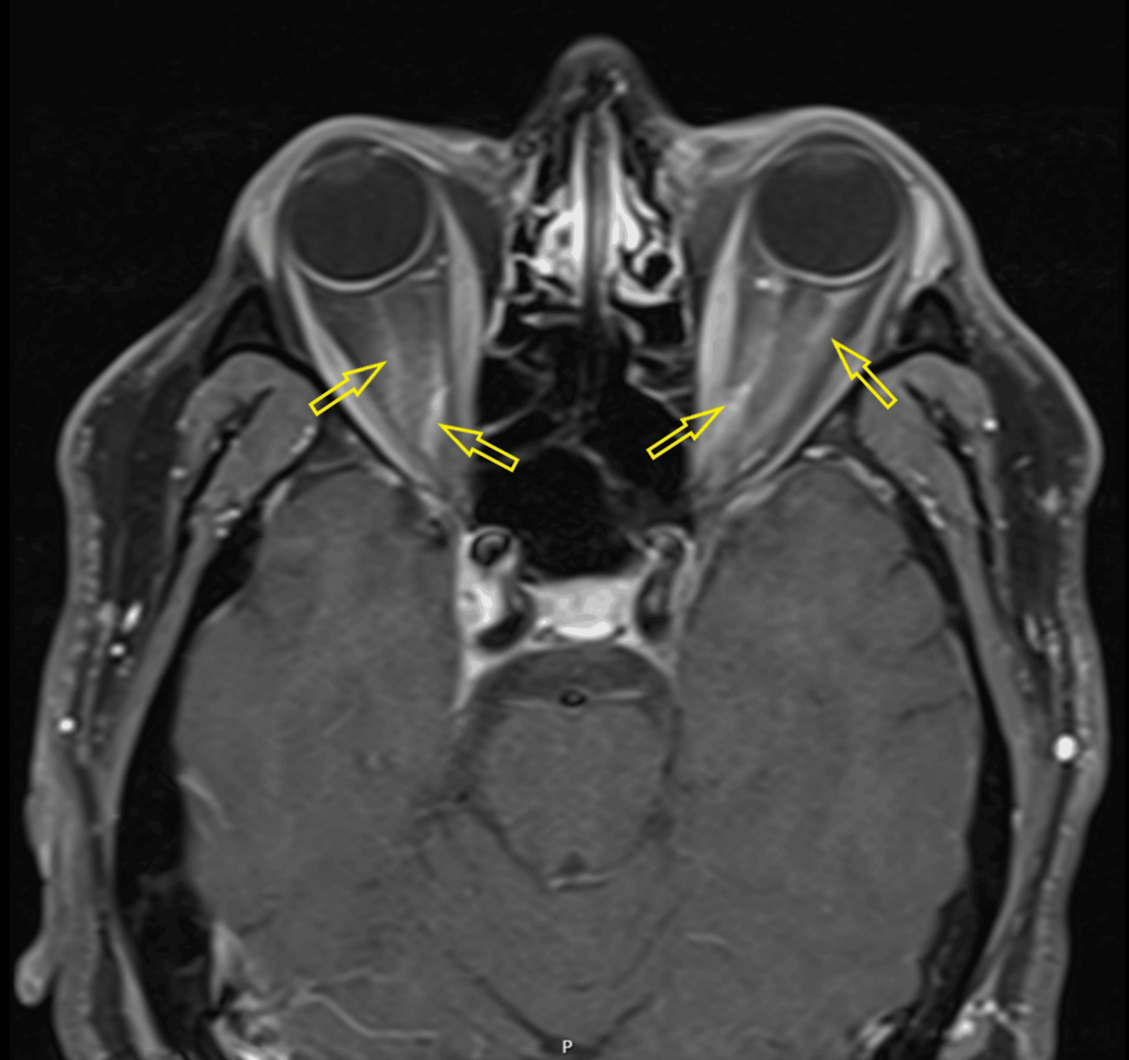
Post-contrast T1-weighted axial MRI demonstrating the contrast enhancement of the optic nerve sheath bilaterally.

**Figure 8:**
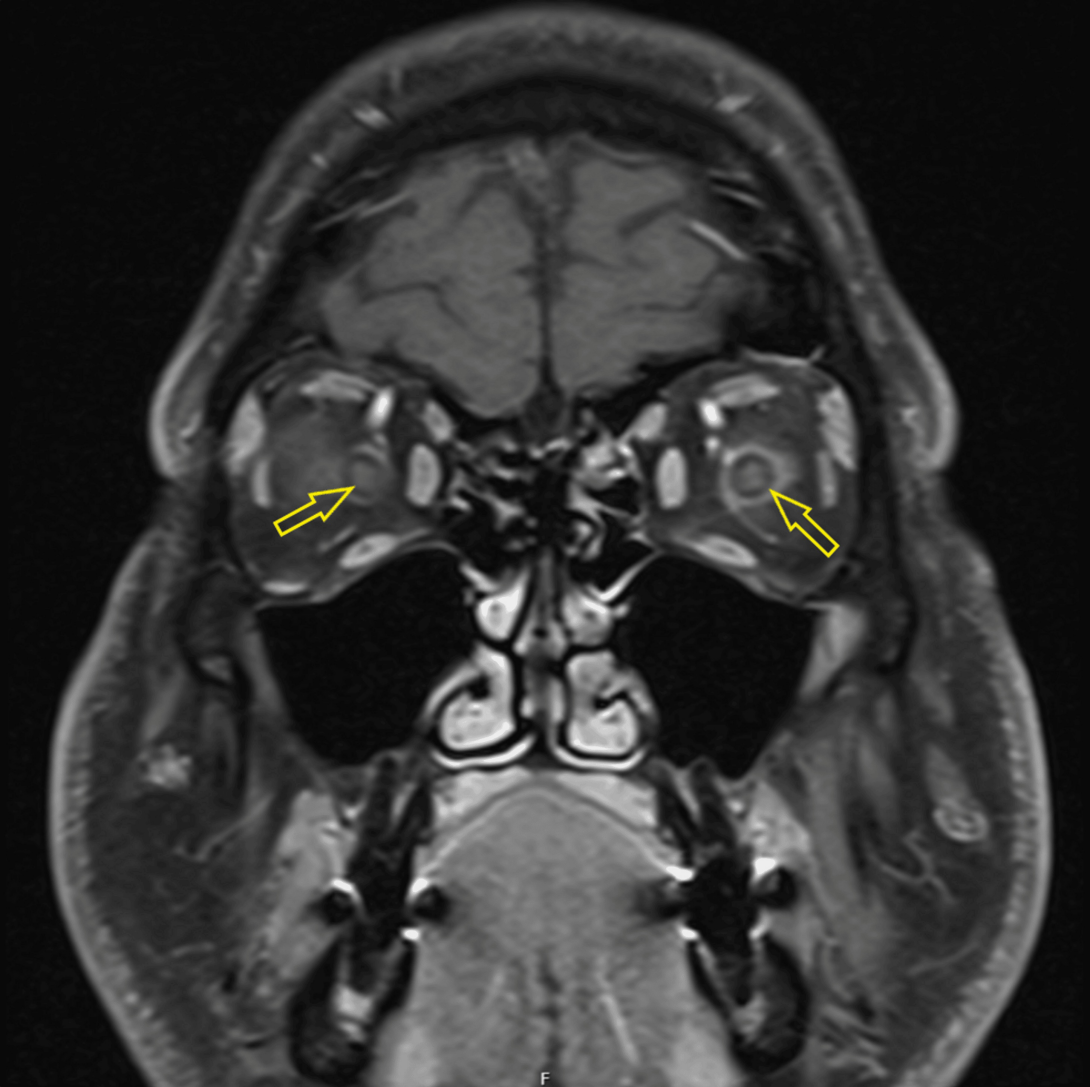
Post-contrast T1-weighted coronal MRI image showing left greater than right optic nerve sheath contrast enhancements and thickening, consistent with perineuritis.

Fundoscopy revealed bilateral optic disc swelling. CT of the head and CT venogram were unremarkable. The CSF sample was clear and not bloodstained, with glucose of 3.1 mmol/L. Protein could not be tested due to an insufficient sample. MRI of the head and orbits with contrast showed increased signal within and around the optic nerves bilaterally, more pronounced on the left, with changes suggestive of optic neuritis and perineural enhancement on both sides, predominantly on the left (Figures 7, 8) [6].

Diagnosis and Treatment

The clinical picture was most consistent with bilateral optic perineuritis [1]. He was started on IV methylprednisolone 500 mg OD for five days, followed by oral prednisolone, and transferred to a tertiary neuroscience center for emergency PLEX. Anti-aquaporin-4 and anti-MOG antibodies were sent. A month later, anti-MOG antibodies came back positive, confirming the diagnosis of anti-MOG antibody-related perineuritis.

Outcome and Follow-Up

He showed a complete recovery from steroid treatment and PLEX and returned to work within two months of onset. A follow-up MRI of the head and orbit, three months after initial presentation, showed complete resolution with no intracranial or orbital pathological enhancement. Both optic nerves remained slightly bright on FLAIR but improved.

Interval anti-MOG antibodies were negative after six months. Steroids were slowly withdrawn. The patient remains on yearly neurology follow-up with an annual check of the anti-MOG antibodies.

Case 4: LETM and rhomboencephalitis

A 51-year-old man with no significant past medical history presented with a gradual-onset headache, initially vertex and retro-orbital, which became severe. He also reported fever, eye pain, difficulty passing urine, and constipation.

Clinical Presentation and Investigations

He presented with acute urinary retention and was catheterized.

In view of his presentation and concern for possible central nervous system involvement, a lumbar puncture was performed (Table 3).

**Table 3:** Cerebrospinal fluid (CSF) sample showing lymphocytic pleocytosis and elevated protein, consistent with a non-bacterial inflammatory or viral etiology.

Parameter	Result	Reference Range (Normal)	Interpretation
Appearance	Clear and colorless	Clear and colorless	Normal
Glucose (CSF)	3.5 mmol/L	2.5-4.4 mmol/L (or >50% of serum glucose)	Normal
Protein	0.94 g/L	0.15-0.45 g/L	Elevated
Red blood cells (RBC)	77 cells/µL	0 cells/µL	Mild traumatic tap or minor bleed possible
White blood cells (WBC)	144 cells/µL	<5 cells/µL	Markedly elevated
Differential count	100% lymphocytes	-	Lymphocytic predominance (suggests viral or inflammatory cause)

**Figure 9:**
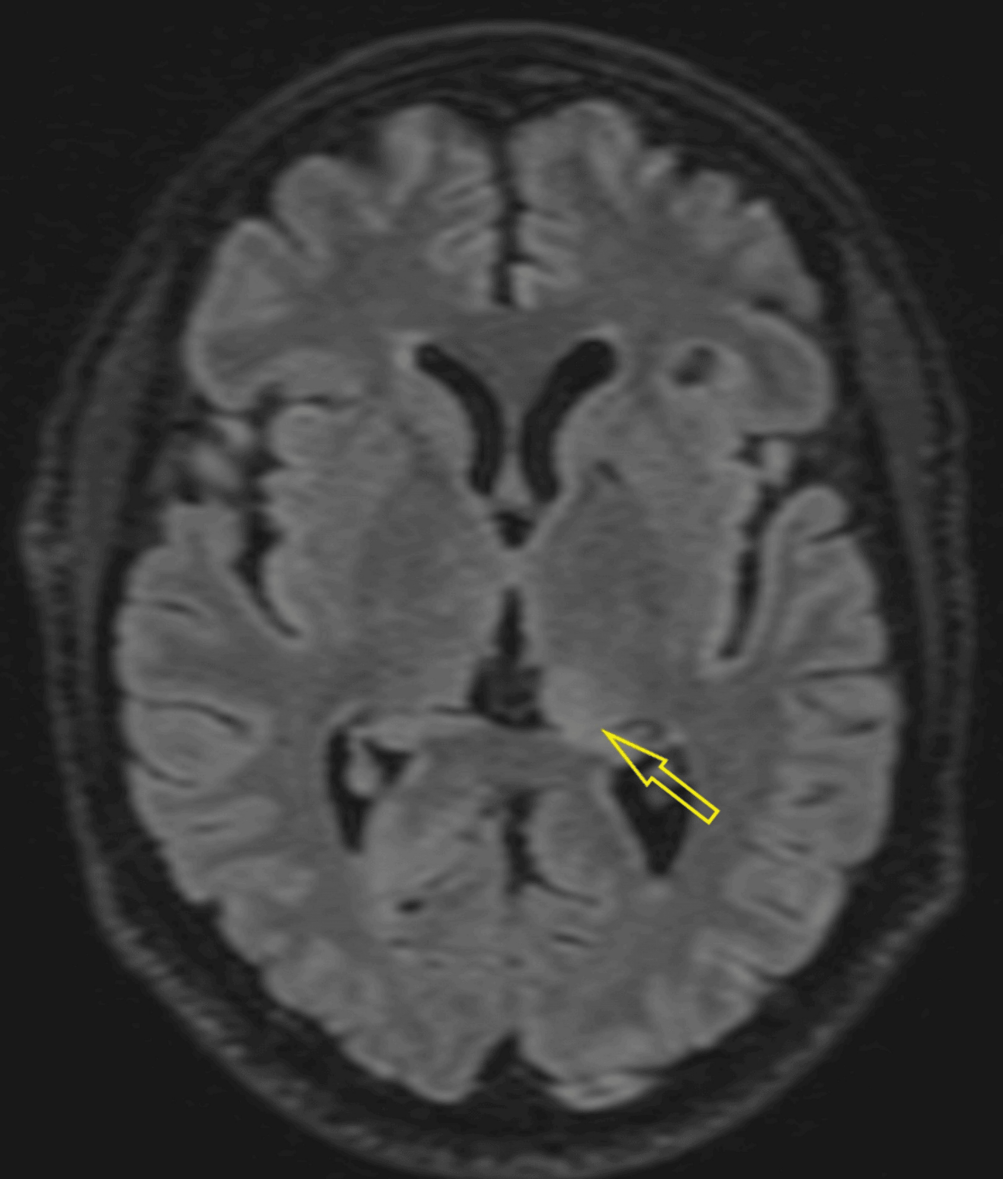
Axial fluid-attenuated inversion recovery (FLAIR) MRI demonstrating a hyperintense lesion in the left dorsomedial thalamus.

**Figure 10:**
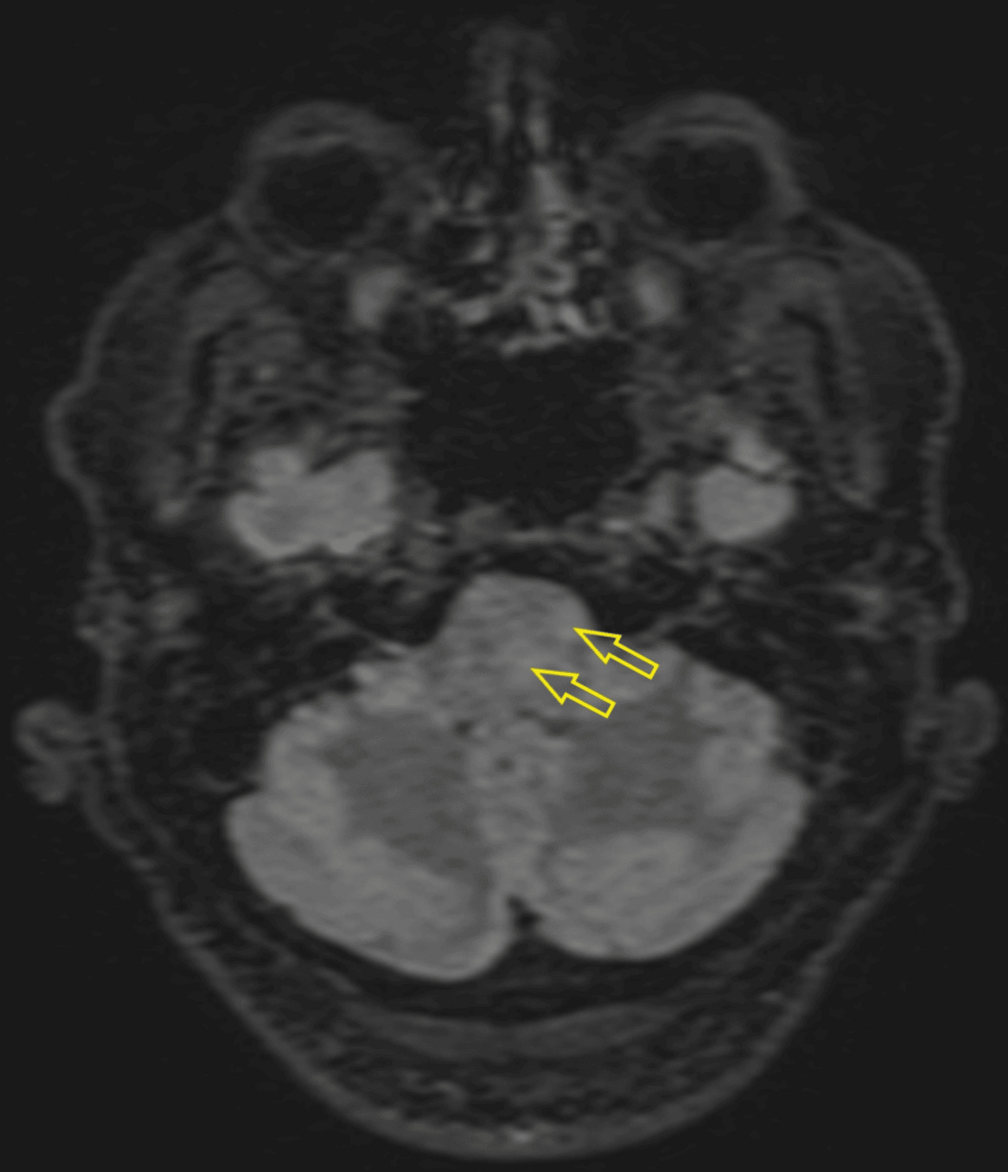
Axial fluid-attenuated inversion recovery (FLAIR) MRI demonstrating patchy hyperintense lesions in the central and left ventral pons.

**Figure 11:**
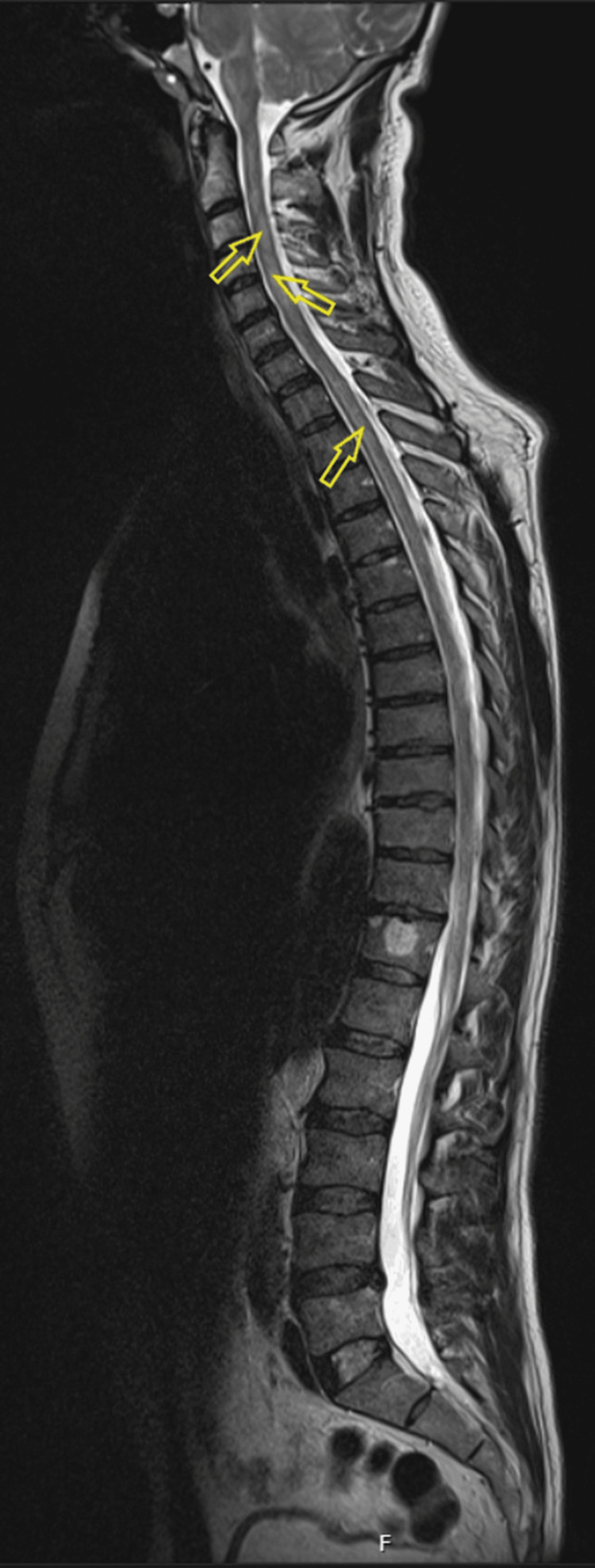
T2-weighted whole-spine MRI demonstrating multilevel short- and long-segment cervicothoracic hyperintense lesions consistent with longitudinally extensive transverse myelitis (LETM).

He progressed rapidly to tetraparesis with diaphragmatic weakness and obtundation. He was admitted to the intensive care unit, five days after admission, for close monitoring with regular forced vital capacity (FVC)/peak flow monitoring. MRI of the head and whole spine with contrast revealed hyperintensities in the bilateral medulla oblongata anteriorly, right anterior midbrain (crus), anterior pons (Figures 9, 10), and throughout almost the entire spinal cord in transverse section (sparing C6-C7). These findings were consistent with a rhombencephalitis and LETM (Figure 11) [7].

Diagnosis and Treatment

On examination, the patient was encephalopathic and dysarthric, exhibiting gaze-evoked nystagmus, spastic tetraparesis, and hyperreflexia, and was catheterized for urinary retention. Expedited testing of anti-MOG Ab returned a positive result (titer of 1:100) within 24 hours. He was treated with pulsed methylprednisolone 1 g daily for five days, followed by oral prednisolone 60 mg daily, and transferred to a tertiary neuroscience center for emergency PLEX.

Outcome and Follow-Up

After PLEX, he showed tremendous neurological improvement, recovering from the complete paralysis of his lower limbs and encephalopathy to the mild/moderate weakness of hip and knee flexors. He was transferred for neurorehabilitation. On follow-up, after six months, he had remarkable neurological recovery but reported residual unpleasant sensory symptoms in his legs and ongoing constipation. Clinical examination was largely normal, with a slight antalgic gait and the diminution of vibration and temperature perception in the distal lower limbs. He was on prednisolone 10 mg OD. Repeat MOG antibodies, eight months after admission, remained positive, indicating a high risk of relapse. His prednisolone was increased to 30 mg OD due to a suspected relapse nine months after the first presentation. He failed to attend a number of follow-up neurology clinic appointments but was eventually started on mycophenolate mofetil, titrated up to 1 g twice daily.

Case 5: LETM

A 29-year-old man, who was previously fit and well, presented with a one-week history of sudden onset of back pain, urinary retention, and perianal numbness, preceded by fever and malaise.

Clinical Presentation and Investigations

**Figure 12:**
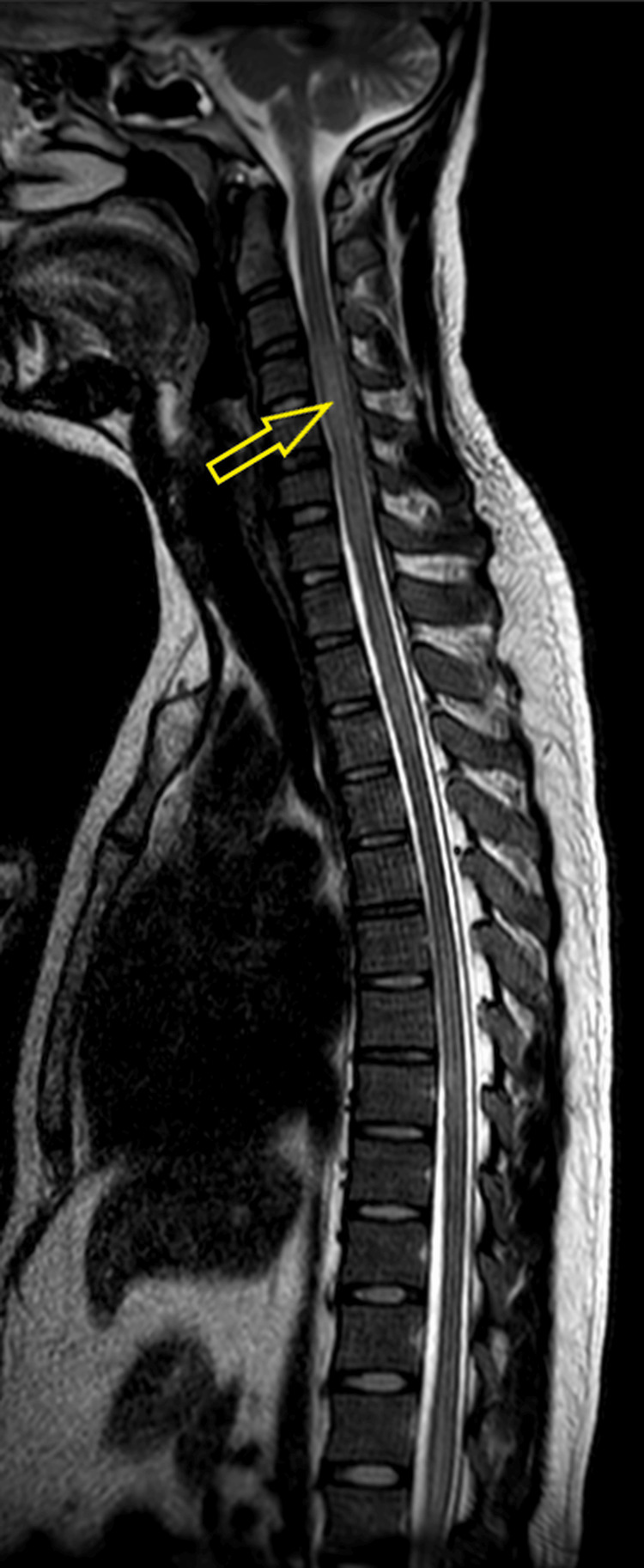
Sagittal T2-weighted MRI of the spine showing intramedullary signal hyperintensity and cord swelling extending from vertebral level C3 to T3, T6 to T9, and T11 to at least L1.

Neurological examination revealed bilateral reactive and equal pupils, 4/5 power in the lower limbs with normal upper limb power, normal tone, and sluggish reflexes in the lower limbs. A bladder scan showed 1200 mL, necessitating catheterization. The patient was urgently transferred to a tertiary neuroscience center. MRI of the brain and spine revealed longitudinally extensive lesions throughout the cord (C4-C7, T1-T4, T6-T10, and T11 to conus) with patchy enhancement, particularly in the cervical region (Figure 12). A few non-specific brain lesions were also noted. The appearance was consistent with LETM, likely inflammatory in etiology. A CT of the thorax, abdomen, and pelvis (TAP) was unremarkable apart from fecal loading and non-specific small pelvic fluid. Infection was excluded.

Diagnosis and Treatment

Based on the MRI findings and the exclusion of infection, a diagnosis of inflammatory LETM was made. The patient received five days of intravenous methylprednisolone followed by five cycles of plasma exchange and was started on oral prednisolone 60 mg. The virology screen was negative. Anti-MOG antibodies returned positive after a month.

Outcome and Follow-Up

The patient was repatriated after 17 days, and prednisolone was slowly tapered down to 20 mg once daily over the next few months. The patient was then transferred to a specialist spinal unit for neurorehabilitation. Unfortunately, this patient was lost to follow-up.

## Discussion

Our five cases here illustrate the diverse clinical spectrum of MOGAD, including isolated optic neuritis and perineuritis (cases 1 and 3), LETM (case 5), and rhomboencephalomyelitis (cases 2 and 4) [1,3,8]. Diagnoses were secured with the serological confirmation of the presence of anti-MOG antibodies [4]. Despite the profound neurological deficits at presentation, all patients benefited from the prompt initiation of high-dose corticosteroids (with slow oral steroid taper) and early emergency PLEX, leading to significant clinical improvement and the resolution of radiological changes typically within 4-6 weeks (Table 4) [1]. This was facilitated by a combination of rapid clinical recognition of disease-defining features and distinct MRI findings (especially optic perineuritis and LETM), along with expedited serological confirmation (often within 24 hours of sample receipt). Expedited serological confirmation was particularly helpful, especially in individuals presenting with encephalomyelitis with pyrexia, head and/or eye pain, and CSF lymphocytosis, which raises the possibility of infectious (tuberculosis, borreliosis, listeriosis, etc.), neoplastic, paraneoplastic, and other neuroinflammatory causes (neuromyelitis optica) [2-4,9]. The initiation of corticosteroid treatment or PLEX may conceivably be delayed for weeks or days while waiting for negative bacterial, mycobacterial, and viral studies.

**Table 4:** A summary of clinical, radiological, and serological findings across the five cases. Abbreviations: M: male; F: female; LETM: longitudinally extensive transverse myelitis; CSF: cerebrospinal fluid; MOG: myelin oligodendrocyte glycoprotein; IVMP: intravenous methylprednisolone; PLEX: plasma exchange; TB: tuberculosis

Case	Age/Sex	Clinical Syndrome	Key MRI Findings	CSF Findings	MOG Antibody	Treatment	Outcome
1	17/F	Optic neuritis and perineuritis	Bilateral optic nerve and sheath enhancement; cortical edema	Lymphocytic, mildly raised protein	Positive (1:1000)	IVMP, PLEX, oral steroids, and mycophenolate	Full recovery
2	27/M	Rhomboencephalitis and myelitis	Brainstem and spinal cord edema	Lymphocytic, ↑ protein	Positive (1:100)	IVMP, PLEX, and anti-TB therapy	Significant recovery
3	52/M	Bilateral optic perineuritis	Optic nerve sheath enhancement	Not available (insufficient sample)	Positive	IVMP and PLEX	Full recovery
4	51/M	LETM and rhomboencephalitis	Extensive brainstem and cord lesions	Lymphocytic, ↑ protein	Positive (1:100)	IVMP, PLEX, oral steroids, and mycophenolate	Partial recovery
5	29/M	LETM	Long-segment cervical and thoracic cord lesions	Not stated	Positive	IVMP, PLEX, and oral steroids	Good initial recovery; lost to follow-up

The relapsing nature of MOGAD is evident in cases 1 and 4, where steroid-sparing immunosuppressants (azathioprine and mycophenolate mofetil) were introduced to prevent further relapses. It is worth noting that case 5 was not started on long-term immunosuppression due to the loss of follow-up, and his MRI showed incomplete resolution, suggesting a high risk of relapse. The decision to withdraw immunosuppression in patients with MOGAD, especially those with persistent positive MOG antibodies, remains a challenge and requires the careful consideration of individual risk factors and disease activity [10,11]. Case 4, despite an initial excellent recovery, experienced a suspected relapse while on a low dose of prednisolone, emphasizing the need for ongoing monitoring and appropriate long-term immunosuppression in high-risk patients.

Several large-cohort studies have highlighted that MOGAD often follows a relapsing rather than a monophasic course [12-14]. The UK population-based study published in JAMA Network Open reported an eight-year relapse risk of over one-third, with younger adults carrying almost double the relapse risk compared to older adults [15]. Similarly, the BMJ Open cohort demonstrated that phenotype at onset influences prognosis: patients presenting with optic neuritis were more likely to relapse than those with transverse myelitis [16]. These findings underscore that relapse risk in MOGAD is both clinically significant and heterogeneous across patient subgroups. Long-term follow-up from the French nationwide study Myelin Oligodendrocyte Glycoprotein Antibody-Associated Disease Observatoire 2 (MOGADOR2) showed that without therapy, relapse risk rises steadily beyond 50% by eight years [14]. Importantly, patients who commenced maintenance immunotherapy immediately after their first attack had a much lower relapse risk, which plateaued at around 20%. Complementary evidence from Neurology Neuroimmunology & Neuroinflammation indicates that early relapses often herald a chronic relapsing course, reinforcing the importance of early treatment decisions [10]. In parallel, immunological insights from Frontiers in Immunology suggest that persistent immune dysregulation and antibody activity provide a mechanistic basis for long-term immunotherapy [11]. Taken together, these studies demonstrate that MOGAD is not reliably monophasic and that relapse is a common, disabling outcome. The evidence strongly supports the early recognition of high-risk patients and the consideration of long-term maintenance treatment strategies to reduce relapse burden and prevent the accrual of neurological disability, points highly relevant to the interpretation of individual case reports. The co-occurrence of MOGAD with other conditions, such as latent tuberculosis in case 2, adds complexity to management and underscores the importance of a comprehensive diagnostic workup.

## Conclusions

This case series emphasizes that MOGAD is a distinct autoimmune demyelinating disorder characterized by a wide range of potentially severe neurological syndromes. Accurate and timely diagnosis relies on recognizing characteristic MRI features and serological confirmation. Early intervention with corticosteroids and plasma exchange (PLEX) is essential to improve outcomes. Due to its relapsing nature, the careful planning of long-term immunosuppressive therapy and close clinical follow-up are necessary. However, definitive guidelines on the optimal duration of immunosuppression remain to be established. Additionally, further research is needed to clarify the predictive value of MOG antibody titers for relapse risk and to refine long-term management strategies.
